# Evaluating the availability and quality of services for lymphatic filariasis morbidity in Ghana

**DOI:** 10.1371/journal.pntd.0010805

**Published:** 2023-06-12

**Authors:** Melissa Edmiston, Solomon Atinbire, Ernest O Mensah, Ernest Mensah, Bright Alomatu, Kofi Asemanyi Mensah, Stephanie Palmer

**Affiliations:** 1 American Leprosy Missions, Greenville, South Carolina United States of America; 2 Ghana Neglected Tropical Disease Program, Accra, Ghana; 3 FHI 360, Durham, North Carolina; University of Ottawa Faculty of Medicine, CANADA

## Abstract

**Background and methodology:**

In districts where lymphatic filariasis (LF) is endemic, the goal is to provide 100% geographical coverage of the essential package of care. Additionally, countries seeking elimination status must document the availability of services for lymphoedema and hydrocele in all endemic areas. To do this, the WHO recommends conducting assessments of the readiness and quality of services provided to identify service delivery and quality gaps. This study used the recommended WHO Direct Inspection Protocol (DIP), which consists of 14 core indicators related to LF case management, medicine and commodities, staff knowledge and patient tracking. The survey was administered in 156 health facilities across Ghana designated and trained to provide LF morbidity management services. Patient and health provider interviews were also conducted to assess challenges and feedback.

**Principal findings:**

The highest performing indicators across the 156 surveyed facilities were related to staff knowledge; 96.6% of health workers correctly identified two or more signs and symptoms. The lowest scoring indicators concerned medication availability, with the two lowest scoring indicators in the survey being availability of antifungals (26.28%) and antiseptics (31.41%). Hospitals performed best with an overall score of 79.9%, followed by health centers (73%), clinics (67.1%) and CHPS compounds (66.8%). The most commonly reported challenge from health worker interviews was lack of medications and supplies, followed by a lack of training or poor motivation.

**Conclusions and significance:**

The findings from this study can help the Ghana NTD Program identify areas of improvement as they seek to achieve LF elimination targets and continue to improve access to care for those with LF-related morbidity as part of overall health systems strengthening. Key recommendations include prioritizing refresher and MMDP training for health workers, ensuring reliable patient tracking systems, and integrating lymphatic filariasis morbidity management into the routine healthcare system to ensure medicine and commodity availably.

## Author summary

This assessment analyzes the capacity of health facilities across Ghana to provide services for lymphoedema, a common manifestation of lymphatic filariasis. Ghana has been working towards lymphatic filariasis elimination over the past two decades. In order to receive official elimination acknowledgement from the WHO, a country must achieve and document a number of indicators including the readiness of health facilities to provide high quality treatment. In this study, the direct inspection protocol (DIP) tool was used to measure health facilities’ capacity to meet key competencies of lymphoedema care related to trained staff, education materials, medications and commodities, and patient tracking. The information collected provides evidence on availability and quality of morbidity management services which can be included in an elimination dossier for lymphatic filariasis. These findings can inform future interventions and approaches to further improve the capacity of health facilities to manage lymphoedema, hydrocele and related complications of lymphatic filariasis.

## Introduction

Lymphatic filariasis (LF) is a vector-borne infection and a neglected tropical disease (NTD). LF is caused by three species of parasitic worms called filariae, with *Wuchereria bancrofti* as the most prevalent worldwide and the only species causing LF in Africa [1]. *W*. *bancrofti* is transmitted from an infected individual via different mosquito species. LF infection may be asymptomatic, but can also lead to acute and chronic conditions, including lymphoedema (swelling of the tissues, primarily the legs), hydrocele (scrotal swelling), and elephantiasis (skin thickening). Adenolymphangitis (ADL), or acute attacks, is also common and can last for weeks causing pain and detrimental disruptions to activities of daily living [2–3].

Like other NTDs, lymphatic filariasis also inflicts a high socioeconomic burden on those affected. Lymphoedema and hydrocele result in long-term disability and often disfigurement, which not only affects an individual’s ability to work and earn a living, but also results in social stigma and rejection [2–3]. An inability or lessened ability to work is exacerbated by the often significant cost of healthcare for LF patients [4]. Furthermore, studies have shown that LF is more common in individuals who have lower socioeconomic status, less education, and poor-quality housing [5,6]. In this way LF is intimately tied with poverty as it poses a great financial burden to the poor and further lessens their social and economic opportunities.

In 2000, the World Health Organization (WHO) launched the Global Programme to Eliminate Lymphatic Filariasis (GPELF). The two primary strategies are 1) to stop the spread of infection through large-scale annual treatment via mass drug administration (MDA), and 2) to alleviate suffering caused by LF through the provision of the recommended essential package of care, which includes: treatment for episodes of ADL, guidance in applying simple measures to manage lymphoedema to prevent progression of disease and debilitating episodes of ADL, surgery for hydrocele, and treatment for infection [7–9]. Morbidity management and disability prevention (MMDP) strategies are critical for alleviating the suffering of affected persons in LF endemic areas. Surgery is needed to address hydrocele, while the severity of lymphoedema can be reduced with appropriate hygiene, skin care, and exercises to support lymphatic drainage. People with lymphoedema must have access to continued care throughout their lives to manage the disease. In addition to preventing disability, education and access to psychological and social support help to fight stigma and support the integration of individuals with LF into society.

WHO’s 2030 Roadmap for NTDs sets the objective to have 90% of endemic counties meet the criteria of validation for LF elimination [9]. In order to be validated for elimination of LF as a public health problem, a country is required to document the following: a) in all endemic areas, the number of patients with lymphoedema and hydrocele (reported or estimated) by implementation unit or similar health administrative unit; b) in all areas of known patients (100% geographical coverage) the availability of the recommended essential package of care; and c) in select designated facilities, the readiness and quality of available services [10].

The Lymphatic Filariasis Elimination Program in Ghana began in 2000, and all endemic districts started MDA of ivermectin and albendazole by 2006. As of 2021, 103 of the 114 districts that were initially endemic for LF have transmission that has been interrupted, as indicated by results from the recommended WHO transmission assessment survey (TAS 1) and achieving the threshold for stopping MDA; 11 hotspot districts remain. The remaining districts have not reached the required level of microfilariae prevalence despite over 10 years of MDA. Since the Ghana LF Elimination Program began, over 74 million people have been treated [11].

In 2019, American Leprosy Missions (ALM) collaborated with the Neglected Tropical Disease Program (NTDP) of Ghana to conduct an MMDP situation analysis as part of USAID’s Act to End NTDs | West Program support to the country. The goal of the situation analysis was to understand the current data availability and quality, the health system’s capacities to manage MMDP, and national program strategies to address LF-associated morbidity as it relates to the WHO essential package of care and LF elimination targets. The situation analysis included an assessment of the health system’s strengths, weaknesses, opportunities, and threats (SWOT) related to MMDP and progress towards LF elimination dossier submission. Findings from the situation analysis revealed that while facilities have been designated and trained to provide lymphoedema management, the readiness and quality of those services have not been assessed. The elimination dossier requires documentation of existing data relates to the readiness of health facilities to provide high-quality treatment for lymphoedema and hydrocele [10]. Therefore, the first objective of this study was to evaluate the capacity of health facilities in Ghana to provide lymphoedema services in accordance with the WHO essential package of care. One recommended methodology for this assessment is the WHO direct inspection protocol (DIP) which evaluates health facilities on six key themes: trained staff, case management and education materials, water infrastructure, medications and commodities, patient tracking system, and staff knowledge [10,12]. A secondary objective was to provide a practical example of how the WHO recommended DIP was implemented, as well as the key findings, to inform other countries preparing for elimination of potential areas of focus.

### Methods

The study used the DIP to conduct a cross sectional survey of designated health facilities across Ghana. The DIP consists of 5 sections: facility information, facility assessment (using the 6 themes), MMDP challenges and feedback, patient interviews and a lymphoedema management demonstration. The first two sections are required, whereas the last 3 are optional and provide additional information. The facility information, facility assessment, MMDP challenges and feedback and patient interview sections were conducted using a standardized survey form and direct observation. The lymphoedema management demonstration was not included.

### Designated facilities

The elimination dossier recommends health facility assessments should be conducted in at least 10% of designated facilities providing services. As of August 2021, health workers in designated facilities in 9 out of the 12 endemic regions in Ghana had been trained to provide MMDP services (3 regions remained to be trained and designated). At the time of this assessment, the NTD Program had designated a total of 1,456 facilities across the 9 regions to provide MMDP services.

### Selected Health Facilities

Of the 9 regions with designated facilities, one region, Greater Accra, was excluded from the assessment because only district hospitals had been trained to provide MMDP services. Therefore, this assessment was conducted in eight regions. Health facilities in the assessment included hospitals, health centers, clinics and community-based health planning and services (CHPS) compounds. A multistage sampling technique was used, starting with the selection of district hospitals and then the rest of the facilities.

#### Selection of district hospitals

At the first level, to ensure that every region had a hospital represented in the sampled facilities, the assessment team purposefully selected at least one hospital per region using random sampling from the list of hospitals. The two regions of Upper East and Upper West were excluded because MMDP training did not include the hospitals, and Bono Region was replaced with an additional hospital in Central Region due to logistical limitations. This selection approach was done to avoid the likelihood of hospitals missing out on the random selection process. The determination of how many hospitals to select was proportional to the number of hospitals trained in the region. Of the total trained facilities, 36 included hospitals; 11 hospitals were selected to participate in the survey, as shown in Table 1.

**Table 1:** Facilities Selected by Region and Facility Type.

Location		Facility Type				
Zone	Region	CHPS Compound	Clinic	Health Center	Hospital	Grand Total
Middle Zone	Bono East Region	9		1	1	11
	Bono Region	6	1	2		9
Northern Zone	Upper East Region		1	7		8
	Upper West Region	3	2	17		22
Southern Zone	Central Region	29	4	13	5	51
	Eastern Region	11		4	2	17
	Western North Region	2		1	1	4
	Western Region	15	2	15	2	34
**Grand Total**		75	10	60	11	156

#### Selection of other health facilities

Because of the variation in facility designation across the country and the uneven distribution (more facilities were designated in the southern part of the country than the north), the country was grouped into three different geographic zones (Northern Zone, Middle Zone, and Southern Zone) to allow for a higher selection ratio for zones with more facility designations. Out of a total 1,456 designated facilities across the country, there are 1,074 in the Southern Zone, 176 in the Middle Zone, and 206 in the Northern Zone.

**Fig 1:**
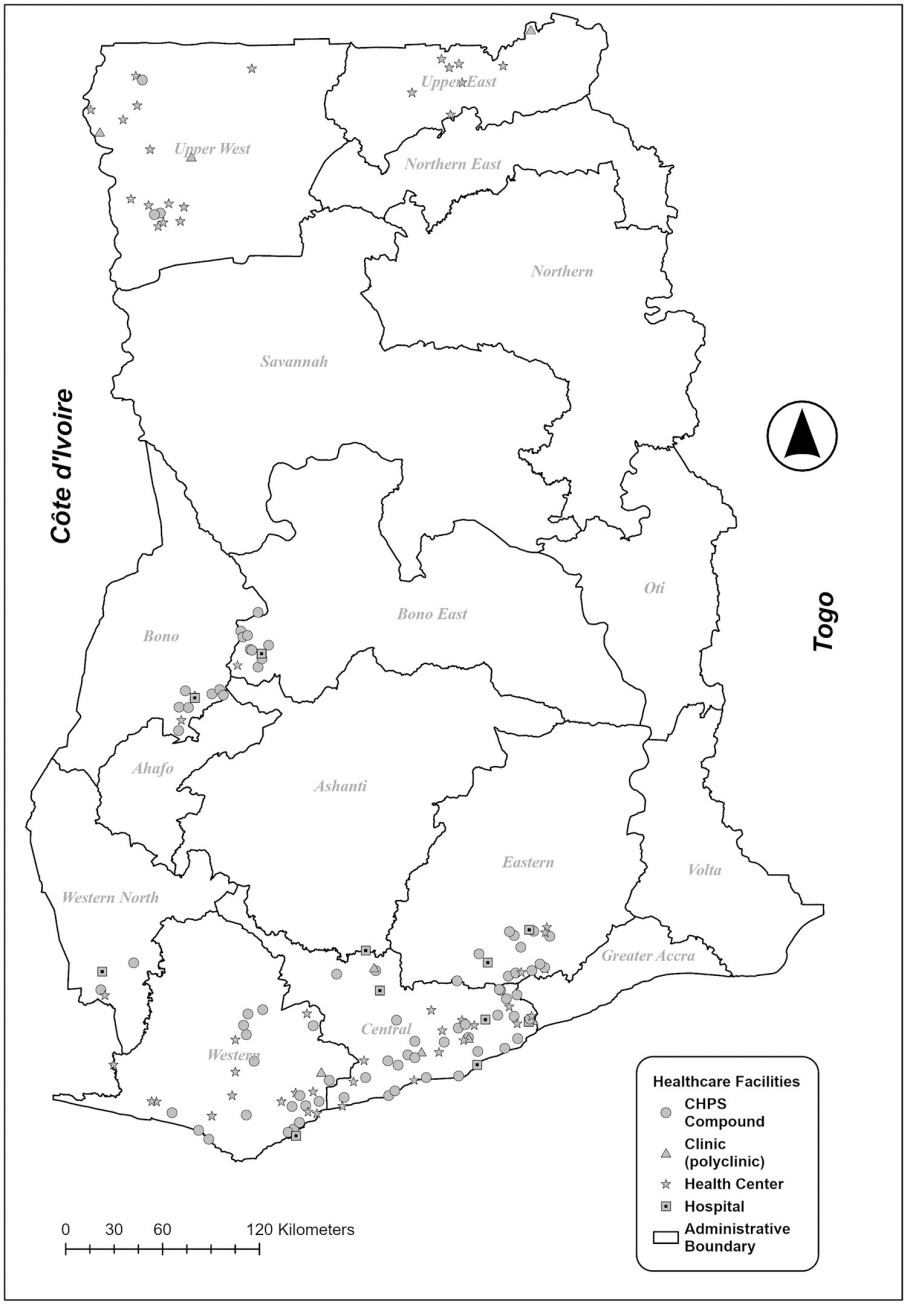
Map of Regions and Health Facilities. Regional boundary data was obtained from https://data.humdata.org/dataset/cod-ab-gha with no changes made. The boundary data is licensed under Creative Commons Attribution for Intergovernmental organizations.

All of the remaining designated facilities in each zone were then pooled together in an Excel file labeled by tabs for each zone. 10% of these facilities were then randomly selected from each zone using SPSS statistical software. In the event that a selected facility was closed or non-operational for any reason, it was replaced with another one in the same sub-district to maintain geographical representation. Field data collectors were instructed with the survey monitors to select the replacement facility from the designated list of facilities in that same sub-district. In total, 156 facilities were selected for the assessment, as shown above in Table 1 by region, zone and facility type and in the map in Fig 1.

### Data Collection

The DIP tool was reviewed and imported into KoboCollect for digital data collection. Minimal changes were made to adjust for the country contexts. The IEC materials indicator was altered slightly from the traditional DIP protocol criteria. This indicator requires that IEC materials are available in the local language. However, after discussions with the NTDP, this was not included in the indicator calculation because very few facilities would have materials available in a language other than English, the official language of the country. Only five facilities indicated they had guidelines targeted at health workers in a language other than English.

Prior to survey implementation, regional level training was held for data collectors on data collection tools, survey questions, facility selection and interview techniques. Those trained included regional disease control officers, regional NTD focal persons, health information officers, and health promotion officers. A total of 39 field data collectors were trained and assigned to various districts based on their proximity and access to selected facilities within the locality. Three national level representatives supervised each zone of the country.

### Health worker and patient interviews

DIP indicators 11–14 are related to staff knowledge. Of the 156 facilities included, 147 had a staff member available who was responsible for lymphoedema management. The survey also included questions for health workers related to MMDP challenges and feedback.

Patients were also invited to participate in interviews based on their availability and willingness. 118 patients were interviewed. Patients were asked to describe the strategies they know for preventing acute attacks and lymphoedema from getting worse. Surveyors were encouraged to listen until no other responses were mentioned. They were also asked questions regarding overall satisfaction and recommendations. For all staff and patients interviewed, verbal consent was obtained.

## Analysis

Data collected from the survey was downloaded from the KoboCollect server and analyzed in Microsoft Excel. The DIP consists of a standard questionnaire with 14 tracer indicators which are grouped into six different domains (Table 2).

**Table 2:** Direct Inspection Protocol Quality Domains and Tracer Indicators.

Domains	Tracer Indicators
1. Trained Staff	1. at least one health facility staff member trained in lymphoedema management in the last two years
2. Case management and education materials	2. at least one guideline for lymphoedema management is present at the health facility 3. at least one information, education, and communication awareness material for lymphoedema management is present at the health facility
3. Water infrastructure	4. the main water for the facility is an improved source, is located on the premises, and is functional at the time of the visit
4. Medicines and commodities	5. antiseptics are present at the facility 6. antifungals are present at the facility 7. antibiotics are present at the facility 8. analgesics are present at the facility 9. at least one supply for lymphoedema and acute attack management is available at the health facility
5. Patient tracking system	10. a system for patient tracking with at least one patient recorded in the last 12 months
6. Staff knowledge	11. clinic staff member able to correctly identify at least one sign or symptom of lymphoedema 12. clinic staff member able to correctly identify at least one lymphoedema management strategy 13. clinic staff member able to correctly identify at least one sign or symptom of an acute attack 14. clinic staff member able to correctly identify at least one strategy to treat a patient with an acute attack

The facilities are scored on a 14-point scale ranging from 0 (lowest performing) to 14 (highest performing). The indicator also receives an overall percentage score based on the points achieved. The scores were summarized by both facility and by quality indicator to identify sub-optimally performing facilities as well as common indicators of concern across facilities.

## Results

Results are presented by region, facility, and indicator. The Western North Region was the highest scoring region based on the average facility indicator scores and the Bono East Region was the lowest scoring region (Table 3).

**Table 3:** Indicator Score by Region.

Zone	Region	Average Indicator Score
Middle Zone	Bono Region	65.9%
	Bono East Region	60.4%
Northern Zone	Upper West Region	66.2%
	Upper East Region	64.3%
Southern Zone	Western North Region	78.6%
	Eastern Region	74.4%
	Central Region	73.4%
	Western Region	70.2%

Table 4 presents the results by both indicator and region. Within each region there was a variation with scores, as well as variation at the district level. S1 Table provides a breakdown of the district and facility level indicator scores.

**Table 4:** Results by Indicator and Region.

	Region							
	Bono East Region	Bono Region	Upper East Region	Upper West Region	Western Region	Eastern Region	Central Region	Western North Region
Indicator 1	81.8%	88.9%	75.0%	72.7%	82.4%	88.2%	84.3%	75.0%
Indicator 2	72.7%	66.7%	12.5%	86.4%	76.5%	41.2%	64.7%	100.0%
Indicator 3	72.7%	100.0%	12.5%	95.5%	85.3%	88.2%	78.4%	100.0%
Indicator 4	54.5%	77.8%	87.5%	77.3%	64.7%	70.6%	72.5%	50.0%
Indicator 5	27.3%	11.1%	50.0%	9.1%	20.6%	58.8%	41.2%	25.0%
Indicator 6	27.3%	0.0%	50.0%	18.2%	8.8%	41.2%	39.2%	0.0%
Indicator 7	54.5%	77.8%	75.0%	22.7%	64.7%	100.0%	98.0%	100.0%
Indicator 8	36.4%	22.2%	75.0%	27.3%	70.6%	82.4%	84.3%	100.0%
Indicator 9	36.4%	0.0%	87.5%	63.6%	76.5%	76.5%	80.4%	100.0%
Indicator 10	36.4%	77.8%	37.5%	90.9%	64.7%	52.9%	31.4%	50.0%
Indicator 11	90.9%	100.0%	87.5%	90.9%	94.1%	82.4%	90.2%	100.0%
Indicator 12	90.0%	100.0%	100.0%	100.0%	91.2%	88.2%	97.8%	100.0%
Indicator 13	100.0%	100.0%	85.7%	100.0%	91.2%	88.2%	95.7%	100.0%
Indicator 14	90.0%	100.0%	100.0%	100.0%	91.2%	82.4%	97.8%	100.0%

Overall, hospitals had the highest DIP scores of all facility types, with an average of 79.9% of indicators met. CHPS compounds were the lowest with an average of 66.8% of indicators met, and were just below clinics, which had an average of 67.1% of indicators met (Table 5).

**Table 5:** Indicator Score by Facility Type.

Facility Type	Total Indicator Score
Hospital	79.9%
Health Center	73.0%
Clinic (polyclinic)	67.1%
CHPS Compound	66.8%

The highest scoring indicator was recognizing signs or symptoms of lymphoedema. Clinic staff members at 91.03% of facilities were able to correctly identify at least two signs or symptoms, demonstrating that health workers are well-trained in basic diagnosis skills in the majority of facilities. This is supported by the fact that the four staff knowledge-based indicators constitute the top highest scoring indicators in the survey. The lowest scoring indicators concerned medication availability, with the two lowest scoring indicators in the survey being availability of antifungals (26.28%) and antiseptics (31.41%). While IEC materials were available in 81.41% of facilities, only five facilities had these materials available in a local language. Each indicator with its average percentage met is available in Table 6.

**Table 6:** Average Direct Inspection Protocol Score by Indicator.

Indicator	Meet Indicator Requirements?	
	Yes	No
1-Trained Staff	82.05%	17.95%
2-Lymphoedema Management Guidelines	66.67%	33.33%
3-IEC Materials	81.41%	18.59%
4-Water Infrastructure	70.51%	29.49%
5-Antiseptics Available	31.41%	68.59%
6-Antifungals Available	26.28%	73.72%
7-Analgesics Available	75.00%	25.00%
8-Antibiotics Available	66.03%	33.97%
9-Lymphoedema Management Supplies	69.87%	30.13%
10-Patient Tracking System	53.21%	46.79%
11-Signs of Lymphoedema	91.03%	8.97%
12-Signs of Acute Attacks	89.74%	10.26%
13-Lymphoedema Management Strategies	89.10%	10.90%
14-Acute Attack Treatment Strategies	89.10%	10.90%

### Indicator-specific results

#### Trained staff- Indicator 1

135 facilities of the 156 included in the assessment (86.5%) reported that at least one staff member currently working at the facility has been trained or retrained in lymphoedema management in the last two years. The most common position trained was nurses.

#### Lymphoedema management guidelines- Indicator 2

68.6% of facilities indicated they had lymphoedema management guidelines targeted at health workers present at the facility. These guidelines were verified through direct observation. Nearly 91% of hospitals had materials available compared to only 66% of CHPS compounds (Table 7).

**Table 7:** Lymphoedema management guidelines’ availability by facility type.

	Hospital	Clinic	Health Center	CHPS Compound
**Yes**	90.91%	70.00%	68.33%	66.22%
**No**	9.09%	30.00%	31.67%	33.78%

#### IEC materials- Indicator 3

127 facilities (81.4%) indicated they had at least one IEC material present at the facility for patient education. The most common materials are shown in Table 8.

**Table 8:** Patient Education Materials Available.

Material Type	Number of facilities
Patient booklets	69
Flip-chart	47
Public awareness poster	39
Morbidity manual	36
Patient leaflets	33
Instruction card	3

#### Water infrastructure- Indicator 4

The water infrastructure indicator consists of three components: facilities where the main source of water is from an improved source as defined by WHO/UNICEF Joint Monitoring Programme (JMP), water source is located on premises, and from which water is available [13]. 95.5% of facilities had an improved water source, and 79.5% of facilities had a water source located on the premises. Table 9 shows the percentage of facilities meeting all three requirements of the indicator.

**Table 9:** Proportion of Facilities with improved source of water on the premises.

Facility Type	Requirements Met?	Percentage
Hospital	Yes	81.82%
Clinic (polyclinic)	Yes	90.00%
Health Center	Yes	81.67%
CHPS Compound	Yes	57.33%

#### Antiseptics available- Indicator 5

Less than one third of all facilities had antiseptics or topical antibiotics in stock in sufficient quantities (Table 10). However, there was variation between facility types, with 73% of hospitals reported having them currently in stock in sufficient quantities, compared to only 30% of health centers and 25% of CHPS compounds.

**Table 10:** Antiseptics or Topical Antibiotic Availability.

Supply Level	Percentage
Currently in stock in sufficient quantities	31.41%
Currently in stock but NOT in sufficient quantities	31.41%
Currently stocked-out	23.72%
Never available	12.18%

#### Antifungals available- Indicator 6

Table 11 shows that only 26% of facilities had antifungals available in sufficient quantities. 63% of hospitals had antifungals in stock in sufficient quantities compared to 19% of CHPS compounds.

**Table 11:** Antifungals Availability.

Supply Level	Percentage
Currently in stock in sufficient quantities	26.28%
Currently in stock but NOT in sufficient quantities	24.36%
Currently stocked-out	29.49%
Never available	19.87%

#### Antibiotics available- Indicator 7

This indicator assesses if facilities have at least one oral or injectable antibiotic currently in stock in sufficient quantities. 66% of facilities had either one or both types of antibiotics available, with roughly one third having neither (Table 12).

**Table 12:** Average Number of LF Supplies.

Facility Type	Number of supplies available
Hospital	5.5
Health Center	3.0
CHPS Compound	2.9
Clinic (polyclinic)	2.6

#### Analgesics available- Indicator 8

75.48% of facilities had analgesic or anti-inflammatory medications currently in stock in sufficient quantities.

#### Lymphoedema management supplies- Indicator 9

The supplies included in the questionnaire are a bucket or basin, soap, towels, gauze or cotton cloth, cold compress, nail clippers, patient hygiene kits and other. The average number of supplies available varied by facility based on the seven included in the survey, with hospitals reporting the highest availability (Table 12).

#### Patient tracking system- Indicator 10

92.3% of facilities reported having a patient tracking system available. Of those that reported “no” or “don’t know,” two thirds were CHPS compounds. Many facilities reported having a patient tracking system, but that no patients have been recorded, resulting in 53% of facilities having both a patient tracking system and recently reported patients.

#### Signs of lymphoedema- Indicator 11

96.6% of health workers participating in the survey were able to correctly identify two or more signs and symptoms of lymphoedema. The most often mentioned symptom was swelling. Table 13 shows the percentage who listed each sign or symptom.

**Table 13:** Staff Knowledge of LF Signs and Symptoms.

Symptom	Percentage
Swelling (irreversible)	78.2%
Skin Folds	76.2%
Swelling (reversible at night)	63.3%
Wounds or entry lesions	53.1%
Knobs on the skin	44.9%
Mossy lesions	40.8%
Acute attacks/ADL	38.1%
Unable to perform daily activities	30.6%

#### Signs of acute attacks- Indicator 12

95.24% of participants were able to identify two or more signs and symptoms of acute attacks. The most common included fever, painful limb, headache, redness of limb, and chills.

#### Lymphoedema and acute management strategies- Indicator 13 and 14

94.5% of health workers were able to identify two or more lymphoedema management strategies and two or more strategies to treat a patient with an acute attack.

### Health Worker Challenges

When staff were asked if the facility faced challenges in providing high quality care to patients with lymphoedema, 82% answered yes. The most commonly reported challenge was lack of medications/supplies, which matches well with the results of the DIP assessment indicators. The second and third most common challenges mentioned were lack of training and poor motivation of providers (Table 14).

**Table 14:** Challenges Reported by Health Workers.

Challenges	Number Reporting
Lack of medication/supplies	105
Lack of training	75
Poor motivation of providers	69
Poor supervision or support	41
Lack of human resources	29
Never encountered a person with lymphoedema	23
Other	18
Was not aware I needed to provide this service	7

### Patient Interviews

Patients were asked to describe the strategies they know for preventing acute attacks and lymphoedema from getting worse. The most common strategy mentioned was hygiene use (86% of patients), followed by elevation, exercise, and wound care. Only two patients reported traditional remedies as a preventative strategy.

Nearly 67% of patients reported washing their affected limb at least once per day. The majority of patients (87%) reported experiencing pain, warmth, swelling or redness of one or both legs, with 50% saying it only happens one or two times per month. However, 17% reported occurrences of more than four times per month. 82% of patients were either satisfied or very satisfied with services received in health facilities. Table 15 presents the results of their response for how services could be improved.

**Table 15:** Patient Recommendations.

Recommendation	Percent
More supplies for patients	72.9%
Increase awareness of program	52.5%
Patient support groups	40.7%
Decrease cost of treatment	31.4%
Improved training for personnel	23.7%
Implement outreach program	22.0%
Increase human resources	10.2%

## Discussion

This DIP analysis assessed the ability of health facilities in Ghana to provide quality lymphoedema services in accordance with the WHO essential package of care. This analysis identified current strengths of lymphoedema care in Ghana, including staff knowledge of diseases and treatments, availability of IEC materials, and to a lesser extent, availability of water infrastructure. This survey also identified weaknesses, including low availability of medications and ineffective patient tracking mechanisms. The DIP methodology does not set benchmarks for each indicator, but rather encourages national NTD Program to assess if an indicator is satisfactory or not and to review which facilities, regions, or specific indicators fell below benchmarks and explore possible solutions and areas for improvement.

Key discussions from the finding involved staff training and knowledge, medicines and commodities, patient tracking systems and patient knowledge and satisfaction. Staff knowledge scored high, but the survey only assessed the knowledge of a specific individual trained. The NTD program is unsure to what extent that knowledge is disseminated to other staff, which is critical for comprehensive capacity. Additionally, those who ranked the highest in staff knowledge were those who received training most recently, indicating that the length of time between training and the time of assessment may have an impact on knowledge. Staff reported lack of training as an overall challenge, so there is a need to ensure training and capacity building is well distributed and maintained.

Availability of medications scored the lowest in terms of overall indicators corroborating the fact that stockouts are a known challenge and there is a need for improved supply chain management as part of overall health systems strengthening [14]. One noted challenge discussed is that health facilities may feel as though LF patients should be treated with medicines and commodities that have been provided by the NTD Program, and do not consider those patients as part of the general patient population. Challenges related to medication availability was identified as a key challenge by health workers as well. Improving patient tracking may be a way to improve supply chain issues and medicine availability by having a better understand of the patient need. The majority of facilities reported having a patient tracking system availability, but only about half of the facilities had patients recorded. Follow-up research is needed to determine if that facility hasn’t seen LF patients recently or if they are just not being entered into the system.

### Next steps

A primary goal of this assessment was to identify gaps and key areas of improvement for prioritization by the Ghana NTD Program to improve services and access to care for persons affected with LF associated morbidity. As they are still several years away from submitting the elimination dossier for LF, there is an opportunity to make improvements in priority areas.

Given the variation in results across districts, facilities and indicators, there is a need to look at detailed results when considering priorities. CHPS compounds and clinics had the overall lowest scores, which is to be expected, but indicates a need to ensure appropriate referral mechanisms are in place and follow-up is conducted.

Moving forward, DIP results suggest that the Ghana NTD Program should continue to provide training on MMDP and to improve health worker knowledge, as well as to prioritize refresher training for health workers. There is also a need to ensure overall integration of LF MMDP into the health system and ensure lymphoedema is considered routine and not specialized, especially as Ghana moves towards elimination. The NTD Program can also explore opportunities for collaboration with other institutions and organizations-for example, to improve WASH infrastructure at certain facilities and explore ways to ensure LF associated medicines are considered in overall supply chain improvements.

In future assessments, there is a need to further probe into health worker knowledge and perceptions of challenges and areas of improvement, as well as patient experiences. Additionally, an assessment of hydrocele services will help to provide a clearer picture of LF services as a whole. The information gathered as part of this DIP assessment is valuable for informing intervention approaches. In the future, more qualitative information will be helpful to fully understand the context of lymphoedema care and to develop appropriate action items as Ghana progresses towards LF elimination. Additionally, the DIP assessment evaluates availability of services, but not whether these services are being accessed and utilized appropriately. Further research is needed is to determine the application, not only the availability, of the recommended essential package of care.

## Supporting information

S1 Table Facility level indicator scores. (XLSX)

S2 Table KoboCollect data collection form. (XLSX)

S1 Data DIP Health Facility Assessment Data. (XLSX)
